# Characterization of Glycan Structures of Chondroitin Sulfate-Glycopeptides Facilitated by Sodium Ion-Pairing and Positive Mode LC-MS/MS

**DOI:** 10.1007/s13361-016-1539-1

**Published:** 2016-11-21

**Authors:** Jonas Nilsson, Fredrik Noborn, Alejandro Gomez Toledo, Waqas Nasir, Carina Sihlbom, Göran Larson

**Affiliations:** 1 Department of Clinical Chemistry and Transfusion Medicine, Institute of Biomedicine, Sahlgrenska Academy at the University of Gothenburg, Gothenburg, Sweden; 2 The Proteomics Core Facility, Core Facilities, Sahlgrenska Academy at the University of Gothenburg, Gothenburg, Sweden

**Keywords:** Chondroitin sulfate, Sodium ion-pairing, Glycoproteomics, Glycopeptides, Oxonium ion, HCD

## Abstract

Purification and liquid chromatography-tandem mass spectrometry (LC-MS/MS) characterization of glycopeptides, originating from protease digests of glycoproteins, enables site-specific analysis of protein N- and O-glycosylations. We have described a protocol to enrich, hydrolyze by chondroitinase ABC, and characterize chondroitin sulfate-containing glycopeptides (CS-glycopeptides) using positive mode LC-MS/MS. The CS-glycopeptides, originating from the Bikunin proteoglycan of human urine samples, had ΔHexAGalNAcGlcAGalGalXyl-*O*-Ser hexasaccharide structure and were further substituted with 0-3 sulfate and 0-1 phosphate groups. However, it was not possible to exactly pinpoint sulfate attachment residues, for protonated precursors, due to extensive fragmentation of sulfate groups using high-energy collision induced dissociation (HCD). To circumvent the well-recognized sulfate instability, we now introduced Na^+^ ions to form sodiated precursors, which protected sulfate groups from decomposition and facilitated the assignment of sulfate modifications. Sulfate groups were pinpointed to both Gal residues and to the GalNAc of the hexasaccharide structure. The intensities of protonated and sodiated saccharide oxonium ions were very prominent in the HCD-MS2 spectra, which provided complementary structural analysis of sulfate substituents of CS-glycopeptides. We have demonstrated a considerable heterogeneity of the bikunin CS linkage region. The realization of these structural variants should be beneficial in studies aimed at investigating the importance of the CS linkage region with regards to the biosynthesis of CS and potential interactions to CS binding proteins. Also, the combined use of protonated and sodiated precursors for positive mode HCD fragmentation analysis will likely become useful for additional classes of sulfated glycopeptides.

**Electronic supplementary material:**

The online version of this article (doi:10.1007/s13361-016-1539-1) contains supplementary material, which is available to authorized users.

**Graphical Abstract:**
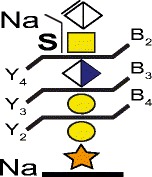
ᅟ

## Introduction

Eukaryotic cells produce various proteoglycans (PGs) that are composed of complex glycosaminoglycan (GAG) chains O-glycosidically linked to Ser residues through a common tetramer (4-mer) linkage region having a GlcAβ3Galβ3Galβ4Xylβ1-*O*- structure [1]. GlcA is glucuronic acid; Gal is galactose; and Xyl is xylose. This 4-mer becomes elongated with repeating GlcAβ4GlcNAcα4 or GlcAβ3GalNAcβ4 units forming either heparan sulfate (HS) or chondroitin sulfate (CS), respectively. GlcNAc is *N*-acetylglucosamine and GalNAc is *N*-acetylgalactosamine. Both chain types are further substituted with specific sulfate groups, which may be evenly distributed along the polysaccharide chains or clustered into unique domain structures. PGs influence a plethora of processes in normal cellular physiology and in embryonic development [2–5]. Several lines of evidence suggest that the underlying activities in many cases depend on selective binding of protein ligands to distinct structural variants of various GAG chains. This topic has been thoroughly investigated, focusing on identifying the specific distribution of sulfate groups along the polysaccharide chains that confer biological activity upon ligand interaction [6–9]. Initiation of the correct biosynthetic pathway has been less studied but early phosphorylation of the linkage region Xyl residue has been proposed to influence the regulation of the GAG biosynthesis [10, 11].

**Figure 1:**
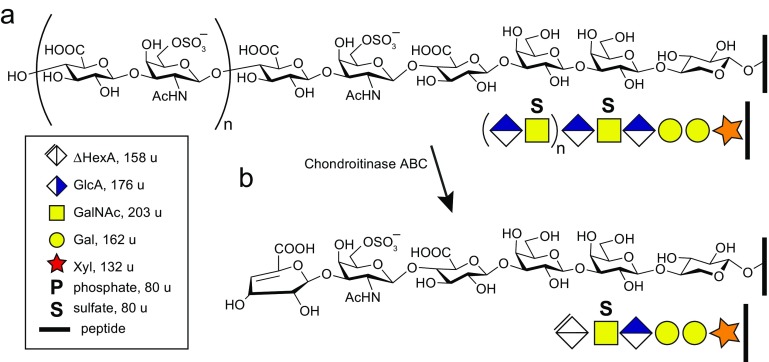
Enzymatic formation of chondroitin sulfate glycopeptides. **(a)** Chondroitin sulfate (CS) is composed of a GlcAβ3Galβ3Galβ4Xylβ1-*O*- tetrasaccharide linkage region attached to the Ser residue glycosylation site. The linkage region is elongated with GlcAβ3GalNAcβ4 disaccharides where n = 10–30. The GalNAc residues may be sulfated, and sulfation may also be present at the Gal and/or GlcA residues. In addition, the linkage region may be substituted with phosphate, fucose, and/or sialic acid, which is described in this paper. **(b)** Chondroitinase ABC treatment results in a hexameric linkage region (6-mer) CS-glycopeptide where the terminal GlcA is dehydrated at C4-C5 (ΔHexA) resulting in a 176 to 158 Da mass-shift. The monosaccharide symbols are according to the Consortium for functional glycomics (http://www.functionalglycomics.org/static/consortium/Nomenclature.shtml). The substitution masses are indicated in the insert

The earlier paradigm for structural analysis of PG involves the removal of the GAG chain from the core proteins and the subsequent characterization of the two remaining components separately [12, 13], thus precluding conclusive evidence regarding site-specific glycan structures, their exact attachment sites, and the identities of the corresponding core proteins. For CS chain analysis, chondroitinase ABC is commonly applied to decompose the CS chains into ΔHexAGalNAc disaccharides, further analyzed by chromatography and MS [14, 15]. ΔHexA (Δhexuronic acid) is a characteristic for a chondroitinase ABC generated dehydration at C4-C5 of GlcA in the CS chain, and is denoted ΔHexA due to the loss of stereoisomerism at C4 and C5 (Figure 1). Although this degradation strategy is informative, the analysis only provides information on the CS polysaccharides in terms of their constituent disaccharides. Further, larger GAG glycans have also been structurally characterized with a focus on the sulfation pattern along the chain [16–20]. Studies aiming at developing effective methods for a more complete characterization of PGs, i.e., characterizing the site-specific glycan structures, including possible structural variations of the linkage region, have only recently come to attention [21–23]. This is likely to be rewarding since methodological advances in other areas of glycobiology, such as the development of glycoproteomic methods for site-specific analysis of protease digested N- and O-glycopeptides, have recently proven to be of importance [24–28].

To obtain integrated glycan-protein information, we recently developed a glycoproteomic approach allowing for the site-specific analysis of CS-proteoglycans [21]. Strong anion exchange (SAX) chromatography was used to enrich GAG-peptides from trypsin-digested human urine, plasma, and cerebrospinal fluid (CSF) samples. Several CS linkage region-substituted glycopeptides (CS-glycopeptides) having a 6-mer saccharide structure were formed after chondroitinase ABC degradation (Figure 1) [21, 22]. High-energy collision dissociation (HCD)-based LC-MS/MS analysis enabled the simultaneous fragmentation and identification of glycan and peptide backbone in an integrated glycopeptide characterization. CS-glycopeptides from bikunin (UniprotKB ID: AMBP_HUMAN) with the 1-AVLPQEEEGSGGGQLVTEVTK-21 sequence were the dominating precursors in these samples. Urinary bikunin was previously known to be modified at Ser-10 (underlined) by a CS-chain comprising 20–60 monosaccharides and 4–9 sulfate groups, but little structural variation of the linkage region has been reported earlier [29–31].

In our analyses, we observed an unprecedented heterogeneity of the bikunin 6-mer glycan with precursor mass differences corresponding to sulfate, phosphate, 5-*N*-acetylneuraminic acid (Neu5Ac), and fucose (Fuc) substitutions [22]. However, during the HCD fragmentation, the sulfate groups were readily abstracted from their saccharide attachment sites precluding the identification of sulfation sites along the 6-mer sequence. This is expected since sulfate group instability during collisional activation is greatly enhanced by mobile protons [13], and released GAG saccharides are therefore exclusively analyzed using negative mode at elevated pH [16, 17]. Moreover, the addition of Na-ions to outcompete remaining protons leads to stabilization of sulfate groups [19, 20].

Negative mode LC-MS/MS of CS-glycopeptides could thus be a possibility in order to specifically analyze the sulfation patterns, but reports so far for negative mode LC-MS/MS analysis of glycopeptides have only shown limited success [32, 33] as they were not very informative with respect to the peptide sequencing. As an alternative, we added sodium acetate directly into the MS-vials and found that the sodium ions stabilized the sulfate groups under positive mode LC-MS/MS conditions, and enabled pinpointing of sulfate groups to the GalNAc and to the outer Gal of the 6-mer [22]. In the present study, we have investigated the optimal Na^+^ concentrations and LC-MS/MS conditions for pursuing successful CS-glycopeptide characterizations. Particularly, we have explored the CS-glycopeptide-derived oxonium ions, using both sodiated and protonated precursors, to gain a deeper structural information of CS-glycopeptide fragmentations. With these analyses we now found even greater structure complexity of the bikunin CS linkage regions than in our two previous reports [21, 22]. This methodological advancement may be generally used to study the linkage regions of other CSPGs, which may assist in elucidating the influence of linkage region modifications for the regulation of CS biosynthesis and eventually to explore functional roles of site-specific glycosylations in proteoglycans.

## Materials and Methods

### Sample Preparation

CS-glycopeptides were enriched from human urine as previously described [21]. The use of de-identified human samples for method development is in agreement with Swedish law and was formally permitted by the head of the Clinical Chemistry Laboratory, Sahlgrenska University Hospital. Briefly, urine samples (8 mL) were lyophilized and reconstituted in 0.02% sodium dodecyl sulfate (SDS) in water (1.5 mL). Samples were desalted (PD-10; GE Healthcare, Uppsala, Sweden) with 0.02% SDS mobile phase, lyophilized and SDS removed by a second PD-10 using water as the mobile phase. The samples were re-lyophilized and in-solution trypsin digested using the Protease Max surfactant trypsin enhancer protocol (Promega, Fitchburg, WI, USA). The digests were diluted with 10 mL binding buffer (50 mM sodium acetate, 200 mM NaCl, pH 4.0) and GAG-substituted peptides were purified using strong anion exchange spin columns (Vivapure, Q-mini H; Sartorius, Göttingen, Germany). A wash solution (0.4 mL, 50 mM Tris-HCl, pH 8.0) was used and bound peptides were gradually eluted (0.4 mL) with three buffers: (1) 50 mM sodium acetate, 0.4 mM NaCl, pH 4.0; (2) 50 mM Tris-HCl, 0.8 mM NaCl, pH 8.0; and (3) 50 mM Tris-HCl, 1.6 M NaCl, pH 8.0. The fractions were desalted (PD-10), lyophilized, and subjected to 1 mU chondroitinase ABC in 10 μL reaction buffer (55 mM sodium acetate, pH 8.0) for 3 h at 37 °C. The samples were finally desalted using C18 spin columns (8 mg resin, Thermo Fisher Scientific, Waltham, MA, USA) and dried. A stock solution of 1.0 M sodium acetate:formic acid (1:1) and 5% acetonitrile in water was used to prepare 10, 100, and 500 mM Na^+^ ion concentrations directly in the MS-vials.

### LC-MS/MS Setup

Glycopeptides were analyzed on a Q Exactive (QE) mass spectrometer (MS) coupled to an Easy-nLCII (Thermo Fisher Scientific) and on an Orbitrap Fusion Tribrid mass spectrometer interfaced to an Easy nanoLC1000 (Thermo Fisher Scientific). Glycopeptides were separated using an in-house constructed pre-column (without pre-column at Fusion system) and analytical column set up (45 mm × 0.100 mm i.d. and 250 mm × 0.075 mm i.d., respectively) packed with 3 μm Reprosil-Pur C18-AQ particles (Dr. Maisch GmbH, Ammerbuch, Germany). An acetonitrile (ACN) gradient was run at 200 nL/min from 7% to 37% B-solvent (ACN in 0.2% formic acid) over 60 or 70 min, with A-solvent of 0.2% formic acid, and then up to 80% B-solvent during 5–10 min. Ions were generated and injected into the MS instrument under a spray voltage of 1.8 kV in positive ion mode. MS scans were performed at 70,000 resolution (QE), 120,000 resolution (Fusion), (at *m/z* 200), with a mass range of *m/z* 600–2000. MS/MS analysis was performed in a data-dependent mode, with the top speed 6 s (QE) or top speed 3 s (Fusion) of the most abundant doubly or multiply charged precursor ions in each MS scan selected for fragmentation (MS^2^) by high-energy collision dissociation (HCD) of a normalized collision energy (NCE) value at levels of 20% and 30%. For MS2 scans an isolation window of 2.5 Da was used and the resolution of detection of fragment ions was 35,000 (QE) and 30,000 (Fusion), respectively. In addition, CID-MS2/MS3 experiments were conducted on the Fusion instrument. The CID-MS2 spectra were collected in the quadrupole for the most intense precursor ion in each full scan. A NCE level of 30% and an isolation width of 5 Da was used. The *m/z* 362.11 ion was used for the MS3 selection.

### LC-MS/MS Data Analysis

HCD spectra originating from CS-glycopeptide precursors were searched for in the LC-MS/MS files by tracing narrow *m/z* regions corresponding to selected fragment ions at the MS2 level (Xcalibur software, Thermo Fisher Scientific). Such fragment ions included monoisotopic masses of *m/z* 362.11 for the [ΔHexAGalNAc]^+^ oxonium ion, *m/z* 486.03 for the [ΔHexAGalNAc + SO_3_ + 2Na – H]^+^ ion, *m/z* 1064.54 for the bikunin Y_0_ [peptide + 2H]^2+^ ion, and *m/z* 1170.55 for the Y_1_ [peptide + Xyl + HPO_3_ + H]^2+^ ion. Fragment peaks were manually interpreted using a mass accuracy threshold of ±0.01 Da. A mass accuracy threshold of 10 ppm was used for precursor assignments. The peptide sequence of the bikunin CS-glycopeptides was verified using a NCE of 30% for the fragmentation of protonated precursors into the b- and y-ions. Lists of b- and y-ions were assembled using “MS-Product” at the protein prospector homepage (http://prospector.ucsf.edu). Extracted ion chromatograms of precursor ions were plotted by tracing the first three isotope peaks in the Xcalibur software.

## Results

### Na^+^ Ions at 100 mM and 500 mM Using a Pre-Column, Did Not Impair the Electrospray Ionization or Chromatography Functionality

**Figure 2:**
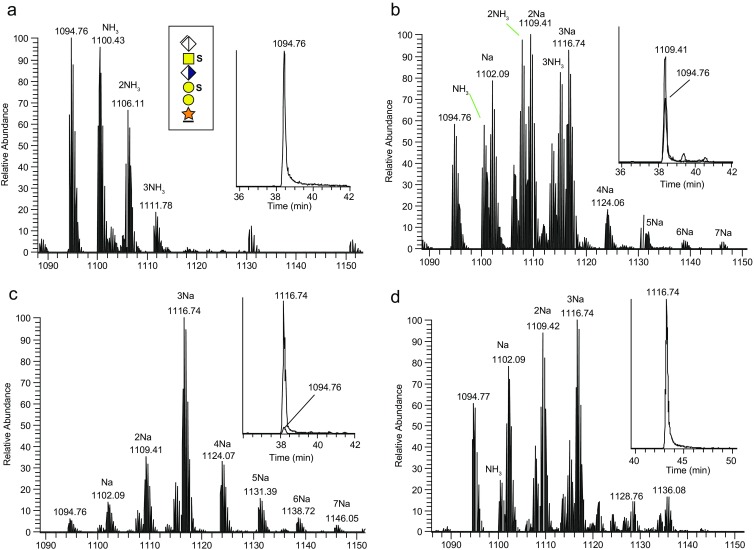
MS1 precursor ions of di-sulfated bikunin CS-glycopeptides at varying concentrations of added Na^+^ ions. **(a)** 0 mM added Na^+^ ions; **(b)** 100 mM Na^+^ ions; **(c)** 500 mM Na^+^ ions; and **(d)** 100 mM Na^+^ ions without the use of a trap column. Extracted ion chromatograms of major precursors are shown in the inserts. The *m/z* values of the largest isotope peaks are annotated. The monoisotopic masses of protonated precursor ions are presented in Table 1

Urinary proteins were cleaved by trypsin and their GAG substituted peptides were enriched by SAX chromatography (Figure 1a), the CS chains were downsized to 6-mer CS-glycopeptides using chondroitinase ABC, and the samples were then subjected to LC-MS/MS analysis using HCD (Figure 1b). The previously described [22] di-sulfated 6-mer (SS-form) of bikunin glycopeptide 1-AVLPQEEEGSGGGQLVTEVTK-21 at *m/z* 1094.76, including up to three ammonium adducts, were the major precursor ions in these samples (Figure 2a). The characteristics of precursor ions of all major CS-glycopeptides described in this paper are presented in Table 1. In the next step, we sought to use Na^+^ ions for ion-pairing of sulfated CS-glycopeptide precursors in order to protect sulfated glycopeptides from losing sulfate group(s) during HCD. Also, it was conceivable that Na^+^ may affect the formation and decomposition of HCD generated saccharide oxonium ions. In order to investigate which Na^+^ concentrations produced sufficient amount of precursor ions without impairing the chromatography and ESI-source functionalities, we conducted LC-MS at increasing Na^+^ concentrations. At 10 mM Na^+^ virtually no Na-adducts were observed for the SS-form, similar to Figure 2a. We then raised the Na^+^ concentration to 100 mM (Figure 2b) and to 500 mM (Figure 2c). At 100 mM Na^+^, a mixture of sodium, water, and ammonium-adducts were observed but at 500 mM the Na-adducts dominated, and the [M + 3Na]^3+^ precursor at *m/z* 1116.74 was the most intense ion. Extracted ion chromatograms (XICs) for the major precursors (Figure 2a, b, c, inserts) showed that the chromatographic peak widths and intensities were not adversely influenced by the addition of Na^+^ to the samples. Without the use of the pre-column, a concentration of 100 mM Na^+^ was sufficient to produce a mixture of [M + 2Na + H]^3+^, [M + 3Na]^3+^, and [M + 4Na – H]^3+^ precursors (Figure 2d). In summary, with the use of a trap-column, up to 500 mM Na^+^ is tolerated; but if no trap-column is used, concentrations of up to 100 mM Na^+^ is sufficient.

**Table 1:**
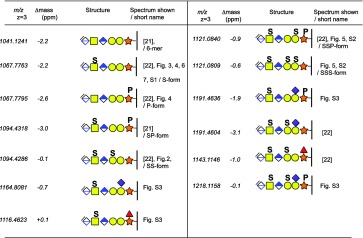
Bikunin CS-Glycopeptide Structures Presented in this and Previous Studies [21, 22]. T﻿he Theoretical *m/z* Values and Accuracies of the Measured Precursor Masses (Δmass in ppm) are Indicated

### Na^+^ Ion Pairing Stabilizes Sulfate Groups Enabling a Detailed Structural Characterization

**Figure 3:**
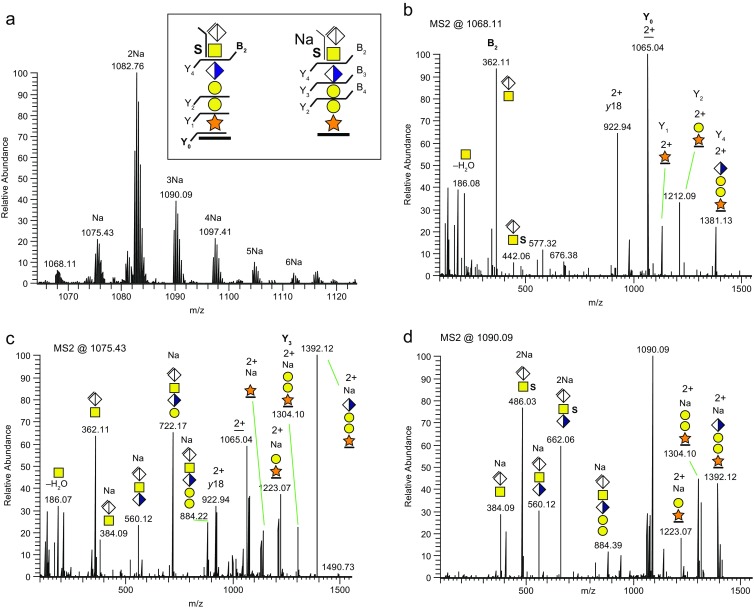
MS1 precursor ions and HCD-MS2 spectra of mono-sulfated bikunin CS-glycopeptides at 500 mM Na^+^ ions. **(a)** The MS1 precursors complexed with 0–6 Na^+^ ions **(b)** HCD-MS2 spectrum of the [M + 3H]^3+^ precursor; **(c)** the [M + Na + 2H]^3+^ precursor, and **(d**) the [M + 3Na]^3+^ precursor. All major fragment ions are annotated with charges >1, *m/z* values, n:o of Na^+^-adducts and glycan structures. The NCE was 20%. The major glycosidic fragmentation sites of protonated and sodiated precursors into the B- and Y-ions are indicated (boxed structures). The *m/z* values of the largest isotope peaks are annotated. The monoisotopic masses of protonated precursor ions are presented in Table 1.The HCD spectra of the corresponding [M + 2Na – H]^3+^ and [M + 4Na – H]^3+^ precursors are displayed in Supplementary Figure 1

The MS1 profile of the SS-form of the bikunin glycopeptide using 500 mM Na^+^ ions was dominated by the [M + 3Na]^3+^ precursor ion (Figure 2c). In contrast, the monosulfated precursor (S-form, Figure 3a), was dominated by the [M + 2Na + H]^3+^ ion, suggesting that one Na^+^ may be predominantly ion-paired to each sulfate group. The HCD spectrum, at 20% NCE level, of the protonated S-form (Figure 3b) showed characteristic glycosidic fragmentation into a desulfated B_2_ ion, at *m/z* 362.11, and into Y_0_, Y_1_, Y_2_, and Y_4_ glycosidic fragment ions (nomenclature according to Domon and Costello [34]). Only trace amounts of the [ΔHexAGalNAc + SO_3_]^+^ oxonium ion at *m/z* 442.06 pinpointed the sulfate group to the terminal disaccharide (Figure 3b). The masses and identities of protonated and sodiated oxonium ions are presented in Table 2.

At 500 mM Na^+^ ions the HCD spectrum of the [M + Na + 2H]^3+^ precursor at *m/z* 1075.43 (Figure 3c) showed additional oxonium ions at *m/z* 560.12, 722.17, and 884.22, corresponding to the annotated structures. The presence of these larger sodiated oxonium ions demonstrated that the Na-pairing protected further decomposition. Furthermore, the Y_3_ ion was a minor ion for the protonated precursor (Figure 3b), but became abundant already at the [M + Na + 2H]^2+^ complexation level (Figure 3c) demonstrating that Na^+^ ion-pairing also protected the glycan core structure from excessive fragmentation. Still, the intensities of sulfated fragments were weak, which also was true for the [M + 2Na + H]^3+^ precursor ion at *m/z* 1082.76 (Supplementary Figure 1A). However, for the [M + 3Na]^3+^ precursor at *m/z* 1090.06 (Figure 3d), sulfated saccharide oxonium ions appearing at *m/z* 486.03 and 662.06 having [ΔHexAGalNAc + SO_3_ + 2Na – H]^+^ and [ΔHexAGalNAcGlcA + SO_3_ + 2Na – H]^+^ compositions were amongst the most intense fragment ions. As the corresponding nonsulfated oxonium ions at *m/z* 384.09 and 560.12 carry only one Na^+^ each, this suggests that the additional Na^+^ ions of the sulfated species are directly paired to the sulfate groups. Also, for the HCD spectrum of the [M + 3Na]^3+^ precursor ion at *m/z* 1090.09 (Figure 3a, d) and the [M + 4Na – H]^3+^ precursor ion at *m/z* 1097.41 (Supplementary Figure 1B) the parent ions were major ions in the HCD spectra, further demonstrating the protection exerted by the Na^+^ ion-pairing.

### Phosphorylation of the Xyl Residue

**Figure 4:**
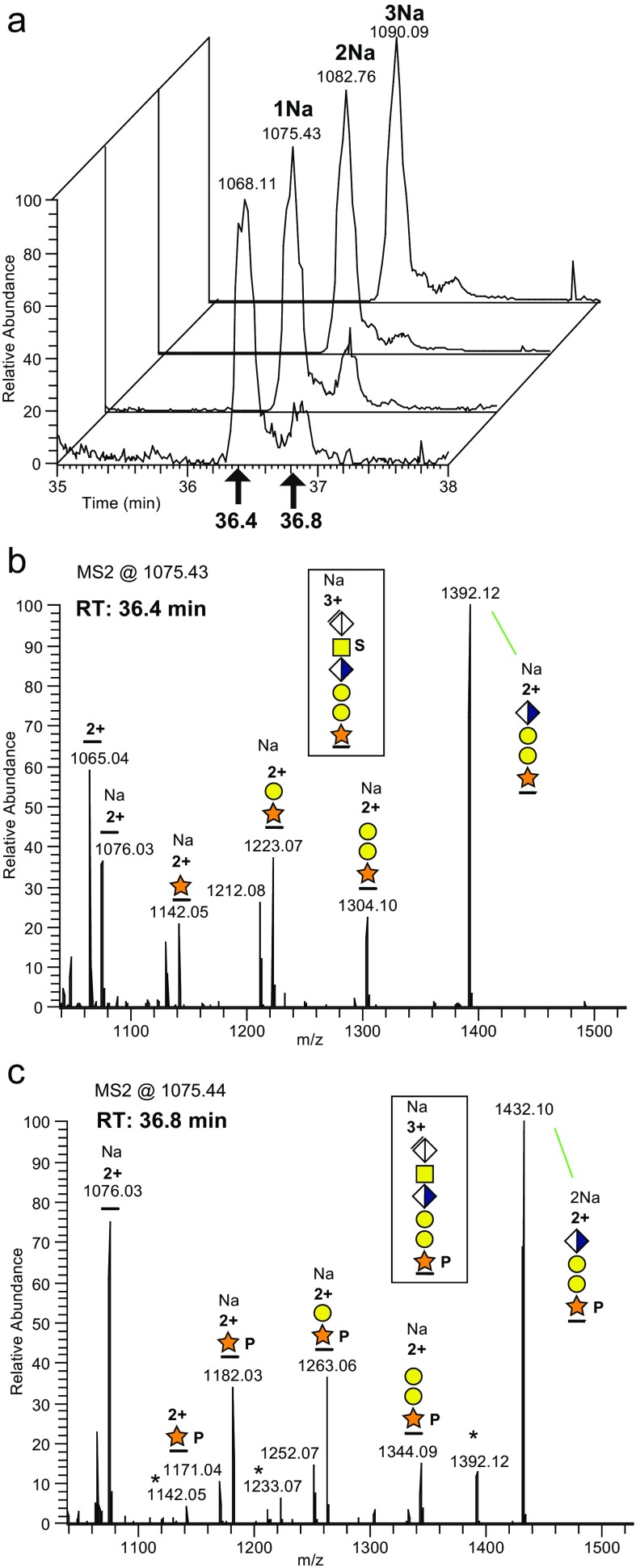
Phosphorylation of the Xyl phosphate **(a)** The extracted ion chromatograms for the [M + 3H]^3+^, [M + Na + 2H]^3+^, [M + 2Na + H]^3+^, and [M + 3Na]^3+^ precursors of the mono-sulfated and the phosphorylated glycoforms of the bikunin 6-mer CS-glycopeptide. The 36.4- and 36.8-min elution time-points are indicated. **(b)** The HCD spectrum, at *m/z* 1000–1500, of the mono-sulfated (S-form) precursor, and **(c)** the HCD spectrum of the phosphorylated (P-form). The NCE was 20%. The full *m/z* 100–1500 HCD spectrum of the [M + Na + 2H]^3+^ is displayed in Figure 3c. The deduced glycopeptide structures are shown boxed. All major fragment ions are annotated with charges >1, *m/z* values, n:o of Na^+^-adducts and glycan structures

The CS-glycopeptide S-form, eluting at 36.4 min (Figure 4a), was followed by a second chromatographic peak eluting at 36.8 min, having nearly identical mass (*m/z* 1075.44 versus 1075.43 for the [M + Na + 2H]^3+^ precursor ions, (see Table 1 where the monoisotopic masses of the corresponding [M + 3H]^3+^ precursor ions are presented). The precursor ions of the later eluting time showed somewhat less affinity for Na^+^ since the [M + 3H]^3+^ and [M + Na + 2H]^3+^ precursors were relatively more intense at 36.8 min, in relation to the corresponding ions at 36.4 min. The *m/z* 1000–1500 regions of the two [M + Na + 2H]^3+^ precursor ions found in the HCD spectra, run at 20% NCE, (Figure 4b, c) demonstrated that the later eluting peak corresponded to a Xyl phosphorylated CS-glycopeptide (P-form) attributable to the presence of a significant Y_1_ ion [peptide + Xyl + HPO_3_ + Na]^2+^ at *m/z* 1182.03 (Figure 4c). Correspondingly phosphorylated fragment ions were found for all precursor ions eluting at 36.8 min including 0–3 Na^+^ ions. Ions marked with an asterisk (Figure 4c) are nonphosphorylated fragments originating from co-isolation of the S-form or from loss of the phosphate group.

### Assigning Sulfates to Both Gal Residues

We previously characterized the di-sulfated SS-form of the bikunin CS-glycopeptide (Table 1) and used Na^+^ ion-pairing LC-MS/MS to pinpoint one sulfate group to the GalNAc and one to the outer Gal [22]. Using protonated precursors, we also described a disulfated CS-glycopeptide that had an additional phosphate group attached to the Xyl residue (SSP-form). The phosphate designation, as opposed to sulfate, was based on the stability of the Xyl phosphate also at 30% NCE for the protonated precursor, but decomposition of all sulfates even at the 20% NCE level. Also, the accuracies of the measured precursor masses, taking the masses of HPO_3_ (79.9663 u) versus SO_3_ (79.9568 u) into account, supported the proposed structure (Table 1).

**Figure 5:**
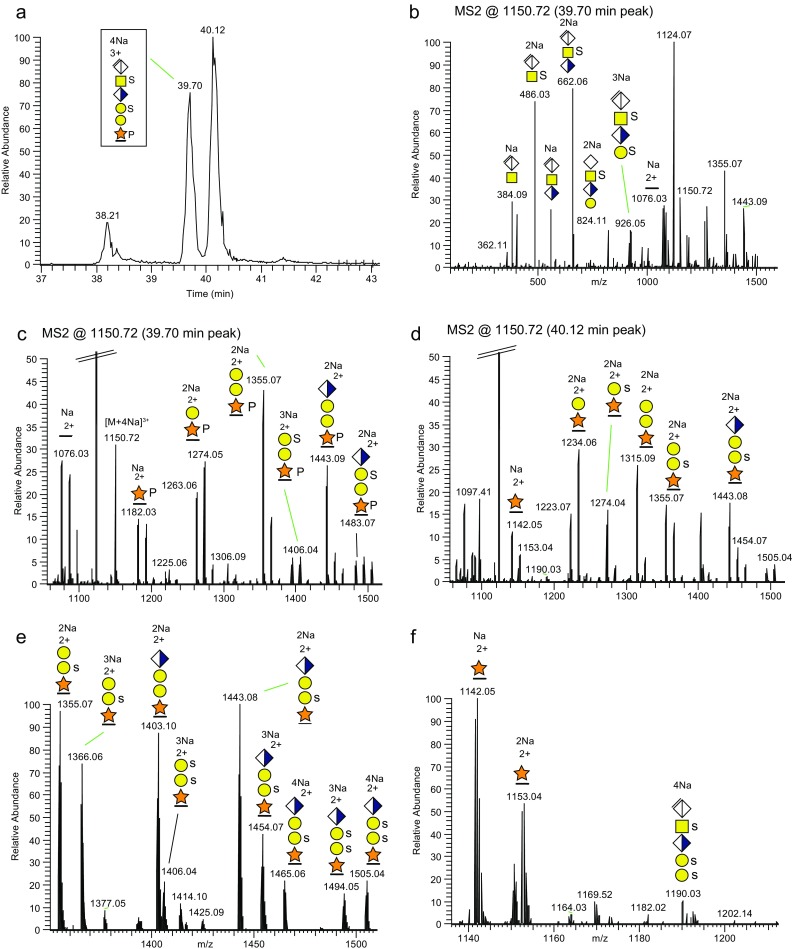
Fragmentation of sodiated precursors of a di-sulfated + phosphorylated glycoform, and a trisulfated glycoform from bikunin. **(a)** The XIC of the *m/z* 1150.29–1151.10 region demonstrating two [M + 4Na – H]^3+^ precursors at 39.70 and 40.12 min. The peak at 38.21 min is not related to these precursors. **(b)** The HCD-MS2 spectrum of the [M + 4Na – H]^3+^ precursor of the disulfated + phosphorylated glycoform (SSP-form), eluting at 39.70 min, and **(c)** the *m/z* 1000–1500 expansion for the 39.70 min spectrum. **(d)** The HCD-MS2 spectrum, at *m/z* 1000–1500, of the [M + 4Na – H]^3+^ precursor eluting at 40.12 min; **(e)** the *m/z* 1000–1500 expansion for the 40.12 min spectrum; and **(f)** the *m/z* 1100–1200 region showing a trisulfated oxonium ion at *m/z* 1190. HCD was performed at a NCE of 20%

Here, we used 100 mM Na^+^ for ion-pairing without the trap-column, to investigate the sulfate/phosphate modifications of the disulfated and monophosphorylated CS-glycopeptide (SSP-form). The [M + 4Na – H]^3+^ precursor (*m/z* 1150.72) eluted at 39.70 min (Figure 5a), and an additional ion, with a slightly higher intensity but nearly the same *m/z*, eluted at 40.12 min. The HCD spectrum of the [M + 4Na – H]^3+^ precursor for the early eluting glycoform (Figure 5b) showed that one sulfate was attached to the GalNAc (B_2_ at *m/z* 486.03, and B_3_ at *m/z* 662.06) and one to the outer Gal residue (B_4_ at *m/z* 926.05). The *m/z* 1000–1500 expansion (Figure 5c) identified an Y_1_ ion at *m/z* 1182.03 corresponding to the Y_1_ ion [peptide + Xyl + PO_3_ + 2Na]^2+^, indicating that the Xyl residue was phosphorylated. No ions corresponding to phosphorylation of the peptide were detected, discounting the possibility of peptide phosphorylation on any of the amino acid residues. The HCD spectrum of the later eluting ion (Figure 5d) showed that it was composed of the bikunin glycopeptide, although lacking the significant Y_1_ ion [peptide + Xyl + HPO_3_ + Na]^2+^ at *m/z* 1182.03 but, instead, including fragment ions corresponding to Y_1_ [peptide + Xyl + Na + H]^2+^ at *m/z* 1142.05 and Y_2_ [peptide + XylGal+2Na]^2+^ at *m/z* 1234.06, indicating that this could be a trisulfated glycoform (SSS-form) of the bikunin glycopeptide.

Further support for the SSS-form came from a significant Y_2_ ion [peptide + XylGal +SO_3_ + 2Na]^2+^ at *m/z* 1274.04 indicating that a sulfate tentatively was additionally attached to the inner Gal residue. Also, for the region of *m/z* 1350–1500, a low intensity Y_3_ ion corresponding to [peptide + XylGalGal + 2SO_3_ + 3Na – H]^2+^ was observed at *m/z* 1406.04 (Figure 5e). Supportive evidence for sulfation of both Gal residues came from a unique B_5_ oxonium ion corresponding to [ΔHexAGalNAcGlcAGalGal + 3SO_3_ + 4Na – 2H]^+^ at *m/z* 1190.03 (Figure 5f). This trisulfation of the 6-mer CS-glycopeptide was partly implied due to the complete decomposition of the sulfate groups during the HCD of the protonated precursor ion (Supplementary Figure 2A), whereas the phosphate group of the SSP-form was stable under these conditions (Supplementary Figure 2B). However, the addition of Na^+^ ions protected the sulfate groups from decomposing and it was then possible to pinpoint the three sulfate groups to their saccharide residues. A theoretical SSSP form of the 6-mer, including Xyl phosphorylation, was searched but could not be detected.

### Neu5Ac Substitution of the CS 6-mer

Inspired by the finding of the SSS-form, we carried on with the manual analysis to search for additional glycoforms. For the protonated LC-MS/MS experiments, a CS-glycopeptide, which was 291 u heavier than the SSP-form corresponding to a sialic acid (Neu5Ac) substitution (Table 1, Supplementary Figure 3A) was indeed found, which is in line with our previously described Neu5Ac substitution of the SS-form [22]. The diagnostic presence of sialic acid-specific oxonium ions [Neu5Ac]^+^ (*m/z* 292.10) and [Neu5Ac – H_2_O]^+^ (*m/z* 274.09) were observed and the presence of [peptide + Xyl + HPO_3_]^+^ at *m/z* 1171.04 but the lack of [peptide + Xyl]^+^ at *m/z* 1130.56 (cf. Supplementary Figure 2) demonstrated that there was a phosphate substitution at Xyl showing typical stability during HCD. The presence of a Y_2_ ion [peptide + XylGalNeu5Ac + HPO_3_]^+^ at *m/z* 1397.62 showed that the Neu5Ac was attached to the inner Gal. In support, the HCD spectrum of the corresponding [M + 3Na]^3+^ precursor (Supplementary Figures 3B and C) showed anticipated fragment peaks, including the Neu5Ac, sulfate, and phosphate modifications. A weak oxonium ion at *m/z* 314.08 corresponding to [Neu5Ac + Na]^+^ (Supplementary Figure 3B) demonstrated that the affinity of Na^+^ to the Neu5Ac carboxyl group was considerably less compared with the sulfate/phosphate group. No precursor ions corresponding to a Neu5Ac substituted SSS-form could be detected, indicating that Neu5Ac and sulfate group substitution of the inner Gal may be attached to the same hydroxyl group. The identification of Neu5Ac substitution of C-6 of the inner Gal residue of the CS linkage region has previously been described [35, 36]. Two additional glycoforms of the bikunin CS-glycopeptide, not previously described, were identified encompassing a sialylated and a fucosylated version of the mono-sulfated S-form (Table 1 and Supplementary Figures 3D and E).

### Fragment Analysis of Oxonium Ions Originating from the [ΔHexAGalNAc]^+^ ion at m/z 362.11

**Figure 6:**
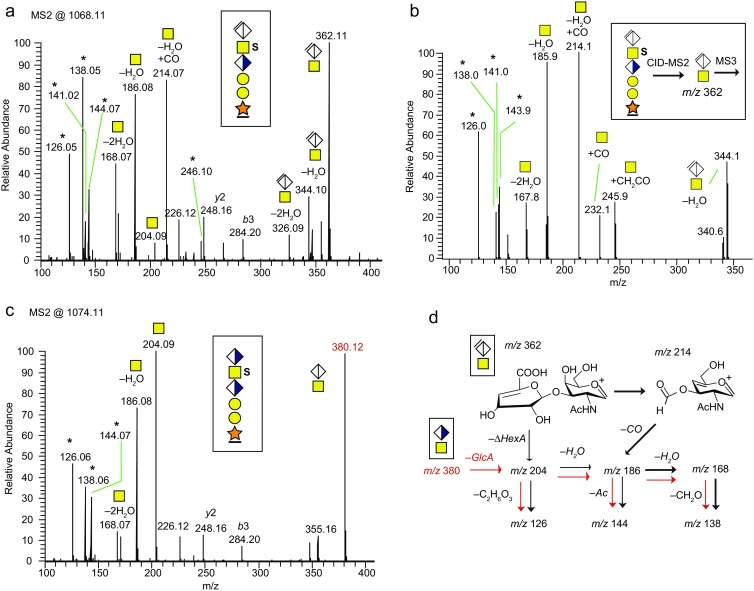
Oxonium ion analysis of protonated CS-glycopeptides originating from the monosulfated glycoform. **(a)** HCD spectrum of the *m/z* 100–400 region showing the oxonium ions. **(b)** CID-MS3 of the [ΔHexAGalNAc]^+^ ion at *m/z* 362.11. **(c)** HCD spectrum at the *m/z* 100–400 region for the mono-sulfated glycoform containing an intact terminal GlcA residue. **(d)** Proposed decomposition pathways for the *m/z* 362.11 and 380.12 (red) ions. All major oxonium ions are annotated with glycan structures and ions marked with * are specified in Table 2. HCD and CID were performed at a NCE of 30%

The HCD spectra of protonated CS-glycopeptides revealed intense saccharide oxonium ions at the *m/z* 100–500 interval (Figure 6a). It was also evident that these oxonium ions, derived from GalNAc residues, gradually disappeared as the number of paired Na^+^ ions increased (Figure 3 and Supplementary Figure 1), demonstrating that Na^+^ ion-pairing prevented the formation of such GalNAc-derived oxonium ions. The prominent presence of the B_2_ ion [ΔHexAGalNAc]^+^ at *m/z* 362.11 is characteristic for a chondroitinase ABC-digested CS-chain since during hydrolysis, the lyase specifically abstracts H_2_O at C4–C5 of the terminal GlcA, thus eliminating the stereoisomerism at C4 and C5 (Figure 1b). Also, a range of oxonium ions resulting from the additional decomposition of the *m/z* 204.09 [GalNAc]^+^ B-ion (i.e., *m/z* 186.08, 168.07, 144.07, 138.05, and 126.05) were present [37]. Additionally, a significant ion at *m/z* 214.07 could potentially originate from the decomposition of the *m/z* 362.11 ion. To further investigate the decomposition of [ΔHexAGalNAc]^+^, we performed CID-MS^2^ of the S-form precursor (*m/z* 1068.2) and then selective CID-MS^3^ of the ion at *m/z* 362.11 (Figure 6b). The CID-MS^3^ spectrum had *m/z* 214.1 as a major ion demonstrating that this ion indeed is formed by the direct decomposition of [ΔHexAGalNAc]^+^. Since *m/z* 214.07 is +27.99 u from *m/z* 186.08, we propose it to be a formylated *m/z* 186 ion, [GalNAc + CO – H_2_O]^+^, formed from cross-ring decomposition of ΔHexA while attached to the GalNAc. The relatively weak ions at *m/z* 232.1 and 245.9, which also were present in the HCD spectra in Figure 6a (*m/z* 232.08 was not annotated here), could be attributed to [GalNAc + CO]^+^ and [GalNAc + COCH_2_]^+^, respectively. Finally, an oxonium ion originating from the terminal ΔHexA losing water [ΔHexA – H_2_O]^+^ at *m/z* 141.0 was observed. As our results suggested that the chondroitinase-related ion at *m/z* 362.11 decomposes into *m/z* 214.07 upon fragmentation, we then analyzed CS-structures, not subjected to chondroitinase ABC, to further study this possibility. Molecular weight cutoff membranes were used for size fractionation purposes to obtain glycopeptides with short CS structures without the use of chondroitinase digestion. In such samples, we detected bikunin 6-mer CS-glycopeptides carrying an intact terminal GlcA residue (Figure 6c). The HCD spectrum showed a strong presence of a B_2_-ion [GlcAGalNAc]^+^ at *m/z* 380.12 but no traces of *m/z* 214.07 or *m/z* 362.11 ions were detected demonstrating that these ions are indeed highly specific for the ΔHexAGalNAc-terminated glycopeptides. The proposed fragmentation pathways of the ions at *m/z* 362.11 and 380.12 (Figure 6d) were partly based on previous reports on the fragmentation of HexNAc residues [37, 38].

### Sulfation of the CS-Glycopeptide GalNAc Residue

**Figure 7:**
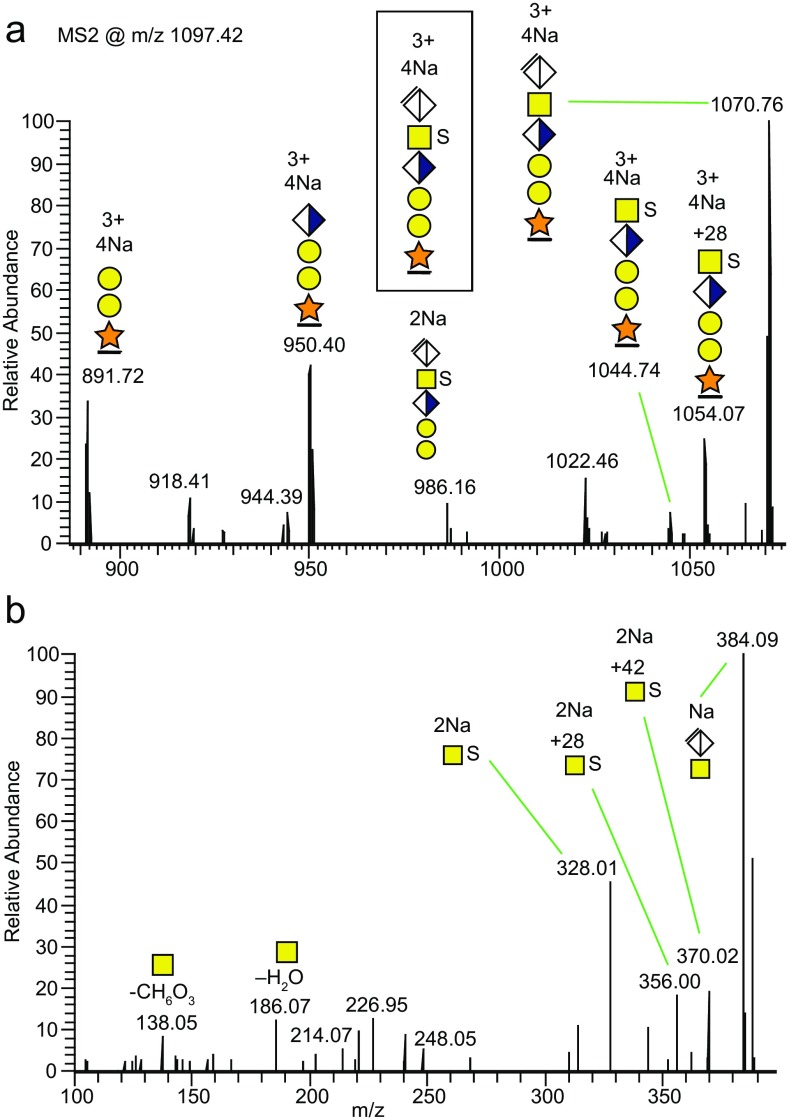
Use of Na^+^ ion-pairing for assigning sulfation of GalNAc in MS2 of mono-sulfated CS-glycopeptide of bikunin. **(a)** The *m/z* 880–1100 region, and **(b)** the *m/z* 100–400 region for the HCD spectrum of the [M + 4Na – H]^3+^ bikunin 6-mer precursor. The NCE was 20%. The full HCD spectrum is displayed in Supplementary Figure 1B

Based on the presence of the *m/z* 214.07 oxonium ion, encompassing the Δ27.99 u CO mass shift, we investigated whether associated mass shifts were detectable in the HCD-induced glycosidic fragmentation of protonated CS-glycopeptides. None were found, but by inspection of the Na^+^ ion-pairing LC-MS/MS experiments we were able to find Δ28 u associated fragments. In a HCD spectrum of the [M + 4Na – H]^3+^ precursor ion of the bikunin glycopeptide S-form at the *m/z* 880–1100 region (Figure 7a), we detected peaks at *m/z* 1054.07, corresponding to the neutral loss of ΔHexA, less the mass of 28 u, and at *m/z* 1044.74, corresponding to the neutral loss of ΔHexA from the precursor ion at *m/z* 1097.42, indicating the presence of a formylated glycopeptide fragment ion. (The corresponding full HCD spectrum is shown in Supplementary Figure 1B). These fragment ions demonstrate that the sulfate group was not attached to the terminal ΔHexA, but was pinpointed to the sub-terminal GalNAc from the intense B_2_ oxonium ion [ΔHexAGalNAc + SO_3_ + 2Na – H]^+^ at *m/z* 486.03 (Supplementary Figure 1B). In addition, in the *m/z* 100–400 region (Figure 7b), the *m/z* 328.01 and 356.00 peaks, corresponding to [GalNAc + SO_3_ + 2Na – H]^+^ and [GalNAc + CO + SO_3_ + 2Na–H]^+^ ions, were observed, also in support of GalNAc sulfation. Furthermore, an ion at *m/z* 370.02 could tentatively be attributed to [GalNAc + COCH_2_ + SO_3_ + 2Na – H]^+^ and is analogous to the ion at *m/z* 246.10, with a probable composition of [GalNAc + COCH_2_]^+^, in the HCD spectra of protonated CS-glycopeptides (Figure 6a, b).

## Discussion

The comparison of HCD spectra of protonated and, to a varying degree, sodiated CS-glycopeptide precursor ions showed that Na^+^ ion-pairing efficiently protected sulfate groups from excessive breakup from their glycan modification positions. Virtually no sulfated glycopeptide fragments or sulfated oxonium ions were observed in HCD spectra analyzed under non-sodiated conditions, but when 1-4 Na^+^ ions were complexed to the precursor ions, sodiated glycopeptide fragments and oxonium ions with retained sulfate groups became abundant. Further, non-sulfated oxonium ions exceeding *m/z* 362.11 and some glycosidic fragment ions (Y_3_) were scarce for the protonated precursors, but became abundant already for [M + Na + 2H]^2+^ showing that Na^+^ ion-pairing also protected the glycosidic bonds from fragmentation, which is in line with a study using Na^+^ ion-pairing and positive mode CID of high-mannose N-glycopeptides [39]. Thereby, the sulfate attachment sites along the innermost 6-mer of the CS-chain could be assigned. Novel 6-mer linkage region structures presented here included a trisulfated glycoform, and one having a Neu5Ac together with two sulfates and one phosphate. The 13 deduced structures (Table 1), presented here and in our previous publication [22], may also be present in the CS structures of additional CS proteoglycans, and should thus be looked for in future CS glycoproteomic studies. The use of Na^+^ ion-pairing to sulfated glycans has previously been used to protect sulfated glycans from decomposing and/or rearranging during collisional activation, mainly in negative mode MS/MS [20, 40, 41]. We now show that the addition of Na^+^ ions may be beneficial for studies of sulfated glycopeptides also in positive mode LC-MS/MS setups.

The analysis of diagnostic oxonium ions, present at the *m/z* 100–400 region of fragmented glycopeptides, has recently appeared as an important tool for assigning the glycan nature of glycopeptide precursors [42–44]. The identification of oxonium ions from protonated CS-glycopeptides (Table 2) are diagnostically important in order to confidently assign the CS nature of the protonated precursor since at elevated NCE levels (>30%), oxonium ions are often the only fragment ions confirming the glycosylated nature of the precursor. At these higher collision energies deglycosylated b- and y-ions dominate [45], which are important for the peptide sequence identification. In contrast, oxonium ions that were abundant in the HCD spectra of protonated precursors were of lower abundance for sodiated precursor ions at [M + 3Na]^3+^ and [M + 4Na – H]^3+^, demonstrating that Na^+^ hindered the production and further decomposition of GalNAc oxonium ions.

**Table 2:** Masses and Identities of CS-Glycopeptide Oxonium Ions

Nominal mass	Monoisotopic mass	Composition
**Protonated oxonium ions**		
126.05	126.0550	[GalNAc-C_2_H_6_O_3_]^+^
138.05	138.0550	[GalNAc-CH_6_O_3_]^+^
141.02	141.0183	[ΔHexA-H_2_O]^+^
144.07	144.0656	[GalNAc-C_2_H_4_O_2_]^+^
168.07	168.0655	[GalNAc-2H_2_O]^+^
186.08	186.0761	[GalNAc-H_2_O]^+^
204.09	204.0867	[GalNAc]^+^
214.07	214.0710	[GalNAc+CO-H_2_O]^+^
232.08	232.0816	[GalNAc+CO]^+^
246.10	246.0972	[GalNAc+CH_2_CO]^+^
274.09	274.0921	[Neu5Ac-H_2_O]^+^
292.10	292.1027	[Neu5Ac]^+^
362.11	362.1082	[ΔHexAGalNAc]^+^
380.12	380.1188	[GlcAGalNAc]^+^
442.06	442.0650	[ΔHexAGalNAc+SO_3_]^+^
**Sodiated oxonium ions**		
328.01	328.0075	[GalNAc+SO_3_+2Na-H]^+^
356.00	356.0024	[GalNAc+CO+SO_3_+2Na-H]^+^
370.02	370.0181	[GalNAc+COCH_2_+SO_3_+2Na-H]^+^
384.09	384.0902	[ΔHexAGalNAc+Na]^+^
486.03	486.0290	[ΔHexAGalNAc+SO_3_+2Na-H]^+^
560.12	560.1223	[ΔHexAGalNAcGlcA+Na]^+^
662.06	662.0611	[ΔHexAGalNAcGlcA+SO_3_+2Na-H]^+^
722.18	722.1751	[ΔHexAGalNAcGlcAGal+Na]^+^
884.23	884.2279	[ΔHexAGalNAcGlcAGalGal+Na]^+^
926.05	926.0527	[ΔHexAGalNAcGlcAGal+2SO_3_+3Na-2H]^+^

For HCD spectra of protonated Galβ3GalNAc-*O*- substituted peptides [38] and the GlcAβ3GalNAc terminated CS-glycopeptide (Figure 6c), the [HexNAc]^+^ ion (*m/z* 204.09) is among the most abundant oxonium ions, but for the ΔHexAGalNAc terminated CS-glycopeptides *m/z* 204.09 was only a minor (<10% relative intensity) ion (Figure 6a). Instead, a unique ion at *m/z* 214.07 was found at a relative intensity of 60%–100% for ΔHexAGalNAc terminated CS-glycopeptides. We have previously proposed that HCD and CID decomposition of [HexNAc]^+^ ions at *m/z* 204.09 into the ion at *m/z* 126.05 takes place via a retro Diels-Alder ring-fragmentation pathway [37]. However, a similar mechanism is not possible for the CS-glycopeptide derived ion at *m/z* 214.07 since its HexNAc ring contains two double bonds (Figure 6d), one at the oxonium oxygen and one from the abstraction of H_2_O from the –OH and –H at C4–C5, disabling a retro Diels-Alder decomposition of the ring. Instead, a probable *m/z* 214 → *m/z* 186 → *m/z* 168 → *m/z* 138 route is favored (bold arrows, Figure 6d).

## Conclusions

The combined use of protonated and sodiated precursor ions of CS-glycopeptides facilitates site-specific characterization of sulfated glycan structures. For protonated precursors, the distinctive oxonium ions at *m/z* 214.07 and 362.11 were diagnostic for all chondroitinase ABC generated ΔHexAGalNAc-terminated structures. The GalNAc-containing oxonium ions formed were further decomposed into smaller ions at *m/z* 126.05, 138.05, 144.07, 168.07, and 186.08. For sodiated precursors of CS-glycopeptides, the formation of oxonium ions in the low mass region was quenched and, instead, sodiated oxonium ions, including sulfates, at *m/z* 486.03 and higher, were the dominating ions. We have demonstrated an extended heterogeneity of the bikunin CS linkage region. The realization of these structural variants should be beneficial in studies investigating the importance of the CS linkage region with regards to the biosynthesis and potential interactions of CS with relevant binding proteins. The combined use of protonated and sodiated precursors for positive mode HCD fragmentation analysis will likely become useful for additional studies of sulfated glycopeptides.

## Electronic Supplementary Material

Below is the link to the electronic supplementary material.

ESM 1 (PDF 732 kb)
