# Supplementary material for: Characterization of Glycan Structures of Chondroitin Sulfate-Glycopeptides Facilitated by Sodium Ion-Pairing and Positive Mode LC-MS/MS
Source: J Am Soc Mass Spectrom. 2016 Nov 21;28(2):229–41. doi: 10.1007/s13361-016-1539-1 (PMC5227003; doi:10.1007/s13361-016-1539-1)
Supplement: Supplementary file 1 — (PDF 732 kb) [file 13361_2016_1539_MOESM1_ESM.pdf]

Supplementary figures

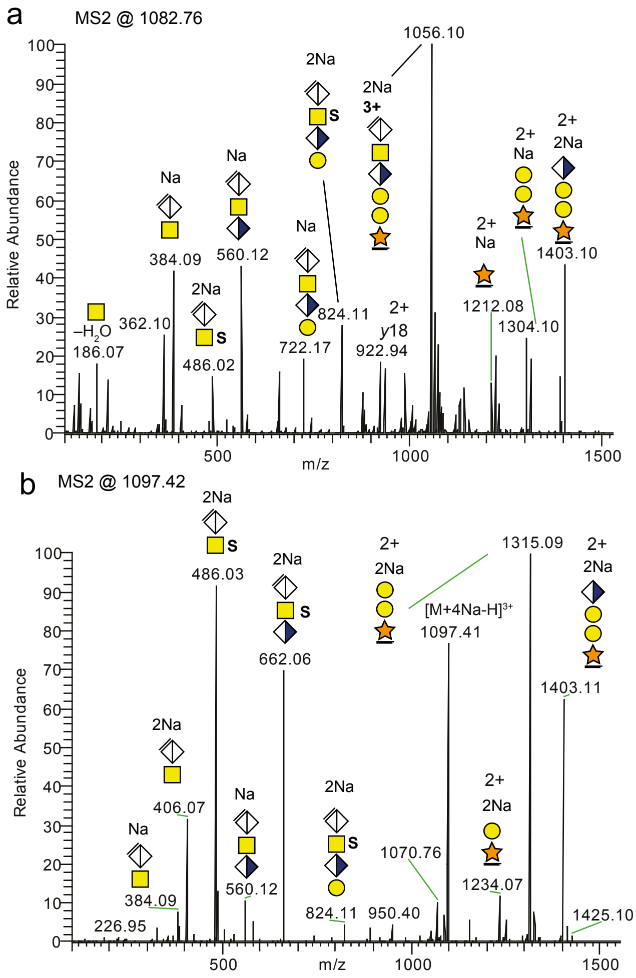

Fig. S1. HCD-MS2 spectra of mono-sulfated bikunin CS-glycopeptides at 100 mM Na^+ ions. (**a**) HCD-MS2 spectrum of the $[M+2Na+H]^{3+}$ precursor and (**b**) the $[M+4Na-H]^{3+}$ precursor. The MS1 survey scan is presented in Fig. 3 of the main text. A NCE of 20% was used. All major fragment ions are annotated with charges > 1, *m/z* values, n:o of Na^+-adducts and glycan structures.

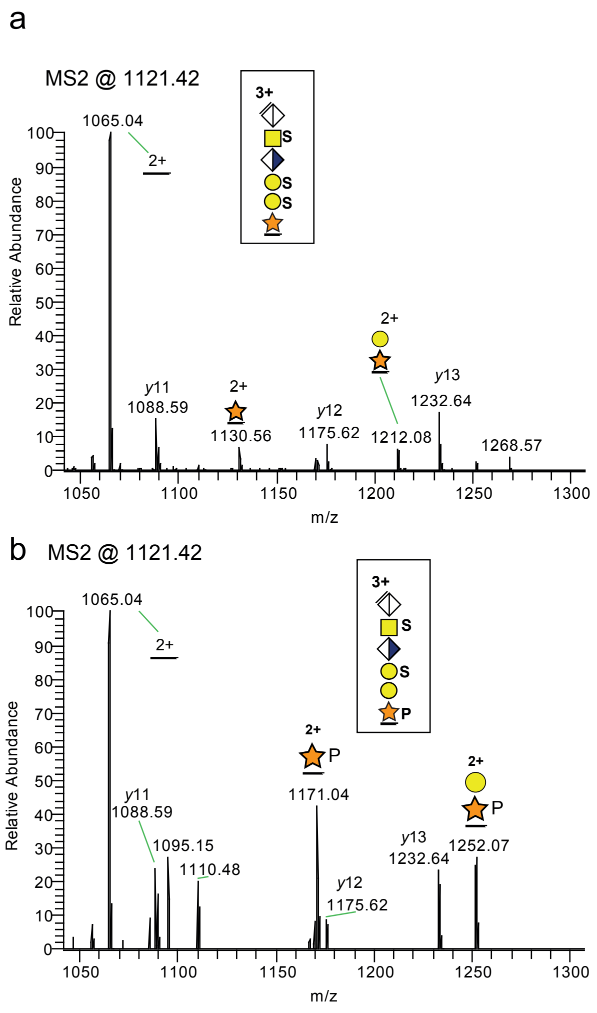

Fig. S2. HCD-MS2 of protonated bikunin CS-glycopeptides. (a) disulfated/phosphorylated (SSP-form) and (**b**) trisulfated (SSS-form). The precursor structures are shown in the boxes. A NCE of 30% was used. Peptide backbone fragmented y-ions, carrying an intact C-terminal, of the AVLPQEEEGSGGGQLVTEVTK sequence are annotated.

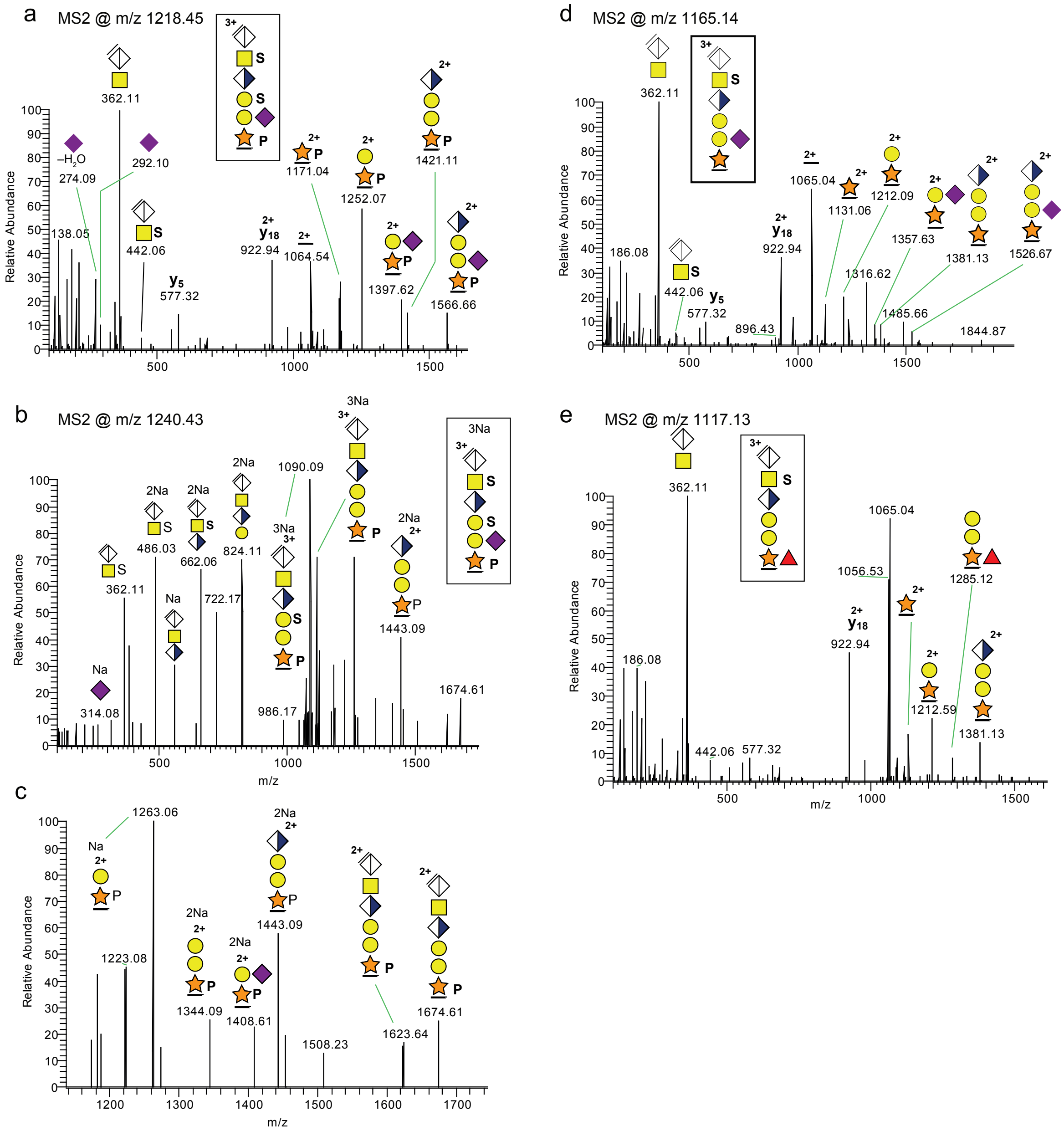

Fig. S3. Additional glycoforms of bikunin CS-glycopeptides. (**a**) HCD-MS2 spectra of a protonated precursor of the sialylated, phosphorylated and di-sulfated glycoform of the bikunin CS-glycopeptide. (**b**) The corresponding $[M+3Na]^+$ precursor and (**c**) the m/z 1100-1700 region. (**d**) A sialylated and mono-sulfated and (**e**) a fucosylated and mono-sulfated glycoform of the bikunin CS-glycopeptide. The precursor ion structures and charge states are indicated in the boxes. A NCE of 20% was used. The *m/z* values of precursor ions are that of the largest isotope peak. The monoisotopic masses of protonated precursor ions are presented in Table 1.
